# Pet Attachment and Wellbeing of Older-Aged Recreational Horseback Riders

**DOI:** 10.3390/ijerph17061865

**Published:** 2020-03-13

**Authors:** Gabriele Schwarzmueller-Erber, Manfred Maier, Michael Kundi

**Affiliations:** 1 Center for Public Health, Medical University Vienna, Kinderspitalgasse 15/1, 1090 Vienna, Austria; manfred.maier@meduniwien.ac.at (M.M.); michael.kundi@meduniwien.ac.at (M.K.); 2 Health Sciences, University of Applied Sciences FH Campus Wien, Favoritenstrasse 226, 1100 Vienna, Austria

**Keywords:** pet attachment, recreational horseback-riding, dog walking, psychological wellbeing, social wellbeing

## Abstract

The aim of the study was to determine if and how emotional attachment to their animal of older-aged (45+) horseback riders affects their physical, psychological and social wellbeing in comparison to dog owners. Overall, 124 individuals 45+ years answered questionnaires about pet attachment and wellbeing. Comparisons were carried out using a general linear model with activity group (rider/dog owner) as the main variable of interest. Horseback riders had no significantly lower pet attachment scores compared to dog owners. Gender differences of pet attachment were found in riders, with women having higher love factor scores. Self-reported mood during activities with the animal was significantly correlated with overall pet attachment, pet love and personal growth by contact with the pet in both, riders and dog owners. We observed no correlation of physical wellbeing during and after the activity with the animal and overall pet attachment in dog owners and horseback riders. Psychological wellbeing during the activity was significantly correlated with overall pet attachment in riders and social wellbeing during the activity in both groups. Recreational horseback riders nearly reach pet attachment scores of dog owners, increasing social and psychological wellbeing in a manner similar to that in dog owners.

## 1. Introduction

To our knowledge, no study has been performed about pet attachment in middle and older-aged horseback riders and its relationship with physical, psychological and social wellbeing. Expanded life expectancy poses new challenges for our society. Increasing life expectancy and, to some degree, longer working periods ask for extending high physical activity into older age in order to promote health. For these ends, activity with pets could be beneficial. 

Participation in sports and exercise improves not only health-related quality of life, but also physical health, such as reducing risk of obesity, cardiovascular diseases, diabetes etc., and psychological health, including reduced mental disorders, better cognitive function, reduced sleeping disorders and stress [1,2,3]. Doing sports, also at older ages, is associated with subjective wellbeing [4]. Regular activities or resistance training enhance muscle function [5], bone health [6] and balance maintenance [7], prevent cardiovascular and metabolic diseases [5], and reduce body fat [8]. 

Not only doing sports, but also walking can already be considered as health promoting physical activity [5]. Walking pet dogs with moderate or medium intensity leads to regular exercise and enables achieving recommended activity levels. Similar to walking with dogs [9,10,11], horseback riding is associated with moderate to high physical activity [12], making it recommendable even for middle and older-aged people.

Wellbeing cannot merely be influenced by physical activity, but also coming into close contact with pets improves owner’s wellbeing. According to the attachment theory of Bowlby, not solely relationships to humans involve high personal attachment, but also to animals [10]. Pet ownership is typically associated with high levels of attachment to the pets [11,13]. Thus, the concepts of human interaction or relationships can be transformed to human-animal interactions [14,15]. Such interactions are especially beneficial in children, in mentally ill patients and in elderly people [16,17,18,19]. Pet attachment is not only observed in service dog owners with physical disabilities [20] but also in other dog owners [10,21,22]. Only few pet owners report no attachment to their pets [23]. The human-animal bond differs between different pet species. People with dogs show the highest attachment scores [15,23,24,25], followed by attachment to cats [10], regardless whether children [13] or adults [26] are concerned. Other studies demonstrated that dog owners are more attached to their pets compared to cat owners [15,24,25]. The longer the ownership, the stronger the relationship to the pets [24]. Attachment to other pets like horses [13], is possible as well. High attachment to horses in horse owners and in clients after horse-assisted therapy was reported [27]. A study showed that the attachment also to horses strengthened with the duration of the ownership [28]. 

Self-esteem and happiness [29,30], physical fitness [31], physical activity [21,32], physical health [10], mental wellbeing [31] and overall quality of life [33,34] improve, and dependency on other people diminished [35] in dog owners. No differences in physical health status in dog versus cat owners was found, while lower attachment in cat owners compared to dog owners was reported [10]. No comparison between dog owners and riders was found in the literature in this respect.

Also, psychological benefits from the relationship to the pet are reported, especially in older adults [36]. Dog owners report fewer depression and anxiety symptoms, and improved social contacts [37] and positive emotions [38,39]. Another study showed that dog and cat ownership was not directly associated with psychological wellbeing [10]. But pet ownership is associated with improved social interactions [40] and social contacts [31,37] thereby enhancing wellbeing especially by its impact on the quality of social contacts [36]. Dog owners talk to each other and other neighbors [23,30], their isolation decreases [30] through dog walking [41] and this results in a feeling of a sense of community and safety [42]. 

Owing to the lack of relevant studies, we aimed to close this gap and to assess pet attachment of middle and older-aged recreational horseback riders in comparison to dog owners for which evidence suggests a relationship between pet attachment and physical, psychological and social wellbeing.

We hypothesize that recreational horseback riders, regardless of gender, show pet attachment scores comparable to dog owners and that pet attachment of recreational horseback riders is associated with physical, psychological and social wellbeing.

## 2. Materials and Methods 

### 2.1. Participants

Horseback riders were recruited with the support of the Equestrian Association of Austria (OEPS); all registered Austrian riding clubs with about 48,500 members [43] received a sheet with details of the study. This sheet was pinned up in the clubs of the different disciplines (dressage, jumping, eventing, western riding, etc.). The search for dog owners was conducted with the help of the Working Dog Sports Association (OEGV) of Austria with about 55,000 members [44]. We addressed family pet dog owners that participate in training courses provided by the OEGV. Overall, 184 riders and dog owners (0.17% of all members) followed the call to participate in the study and were contacted by mail or phone. A face to face screening conversation or a telephone screening call was conducted to confirm the inclusion and exclusion criteria.

Inclusion criteria were being older than 44 years, a recreational horseback rider or dog owner for at least one year and an Austrian citizen. Exclusion criteria were being physically unable to perform horseback riding or non-ownership of a dog and owning both, dogs and horses. 

All participants agreed to spend one hour answering the questionnaire and signed a written informed consent form. 

This study was exempted from ethics approval (IRB of lower Austria: date of submission 18.07.2014, date of waiver 22.07.2014), but was carried out according to the Helsinki Declaration of 1964 [45] and its amendments.

### 2.2. Data Collection and Management

The 14-page questionnaire in German language and the informed consent form were sent to all eligible persons by e-mail or postal mail. During the data collection period, 127 individuals returned the questionnaire (response rate 69%). Participants were requested to fill in the questionnaire directly after performing their hobby (riding, dog playing/”working”), because mood and feelings had to be answered for the period of their activity with the animal.

All questionnaires were checked for completeness and signature of consent form. Questionnaires were numbered for anonymization. Then the data were manually transferred to SPSS Statistics version 24 (IBM Corp., New York, NY, USA).

Overall, 124 individuals (69% of 184 persons who responded to the call) fulfilled the inclusion/exclusion criteria. All of them (95 females, 29 males) consented and took part in the study (*n* = 67 riders, *n* = 57 dog owners).

### 2.3. Questionnaires

The total survey consisted of four sections and was administered in German. For evaluation of pet attachment, the Life-Impact Scale PALS-35 [46] was used. This questionnaire with 35 items assesses the attachment to pets, positive and negative aspects of relationships with pets and the impact of pets on their owners. It consists of four factors which measure “love” (17 items), “regulation of emotions” (9 Items), “personal growth” (5 Items), and “negative impact” (4 Items) [46]. Statements are rated using a five-point Likert scale from 1=not at all to 5=very much. Negative items (4,11,16,29) were reverse scored. For the different scales the item mean was calculated. Internal consistency is excellent with a Cronbach’s alpha of 0.907 [46]. The evaluation of internal consistency within the current study shows a Cronbach’s alpha of 0.934 for the total PALS with 35 items, α = 0.894 for the “love” factor, α = 0.877 for the “regulation” factor, α=0.811 for the “personal growth” factor and α = 0.485 for “negative impact” factor. 

To evaluate self-reported general habitual wellbeing of participants, the “Fragebogen zum habituellem Wohlbefinden” FAHW 12 (questionnaire for habitual wellbeing [47], a 12-item questionnaire using a 5-point Likert scale: 1 = certainly not to 5 = yes, exactly) was used. The questionnaire consists of three subscales, each with four items, assessing physical, psychological, and social wellbeing. It shows a good internal consistency, i.e., Cronbach’s α = 0.85 [47]. The evaluation of internal consistency within the current study revealed a Cronbach’s alpha of 0.80 for the total FAHW 12, α = 0.82 for physical wellbeing, α = 0.63 for psychological wellbeing, and α = 0.53 for social wellbeing.

To assess mood during/after the activity with the animal, a single Kunin (face) item [48] ranging from A = 7 points to G = 1 point according to Andrews and Withey (1976) [47,49] was used. Participants were requested to answer the question “Which face illustrates best how you felt during and feel now after riding/dog playing or dog walking?”


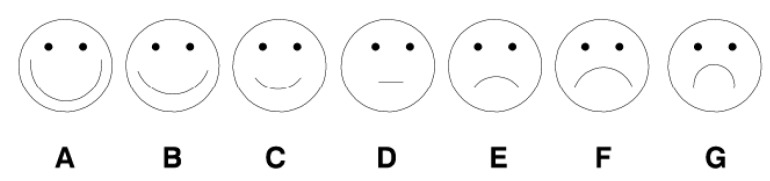


Another part of the questionnaire with 28 items, created by us, was designed to assess the influence of the activity with the animal on wellbeing. Study participants were requested to fill in this part of the questionnaire directly after performing their hobby (riding, dog playing/”working”) for two time periods: the current time (“after activity”) and the time period when they performed the activity with the animal (“during activity”). Internal consistency of these parts of the questionnaire was good for the total scales with Cronbach’s α = 0.80 (both, during and after activity) and moderate for the subscales (Cronbach’s α: 0.56–0.66). (See supplement S1).

Another part of the 14-page questionnaire was dedicated to demographic data of participants, like gender, age, weight and height (calculation of BMI), education, and living conditions, which were used in the general linear models to address possible confounding and also to check comparability of groups. 

### 2.4. Statistical Analyses

Descriptive statistics were used to characterize the study population with means and standard deviations for metric data and numbers and percentages for categorical data. For group comparison chi-square tests and Mann-Whitney tests were applied, as appropriate. 

To generate general habitual wellbeing scores (FAHW 12), the sum of responses to the items with a score ranging from −18 to 25 and the sum of responses to the items representing each subscale were computed. Negatively polarized items (3,4,5,9,10,12) were subtracted from positive ones (1,2,6,7,8,11). The same procedure was applied for the three subscales of physical, psychological and social wellbeing. The higher the score, the higher the self-reported subjective wellbeing [47]. Average sum score reference levels for healthy people are between 10 and 13, above average from 14 to 16 and strongly above average 17 and above [47]. 

To evaluate the rating of subjective wellbeing during and after horseback-riding or playing/working with the dog, the sum score of scales was computed for each subscale: physical, ranging from 0 to 10, psychological and social wellbeing, ranging from 0 to 9.

For the generation of pet attachment scores, the mean of responses to the items ranging from 1 to 5 was computed for the total scale and for the subscales “love” “regulation” “personal growth”, and “negative impact”. For all subscales—except negative impact—high pet attachment is associated with higher scores [46]. 

For assessing the influence of predictors on pet attachment, a general linear model with the possible confounders gender, age, BMI (calculated from weight and height), marital status (fixed = married or living with a partner; or no partnership) and education (more or less than twelve years) was performed. Normality of residuals was assessed by Kolmogorov-Smirnov tests with Lilliefors’ corrected *p*-values and homogeneity of variances by Levene’s tests. Bonferroni-Holm correction was applied for multiple endpoints.

To determine if pet attachment levels influenced the pattern of wellbeing, a correlation analysis was performed using Spearman’s rank correlation. All tests were done by SPSS version 24. For all tests statistical significance was set at 5% (*p* < 0.05).

## 3. Results

### 3.1. Sample Characteristics

Most of the participants were female (riders: 88%, dog owners: 63%). The age of horseback riders ranges from 45 to 82 years, and of dog owners from 45 to 80 years (Table 1).

### 3.2. Pet Attachment

**Figure 1:**
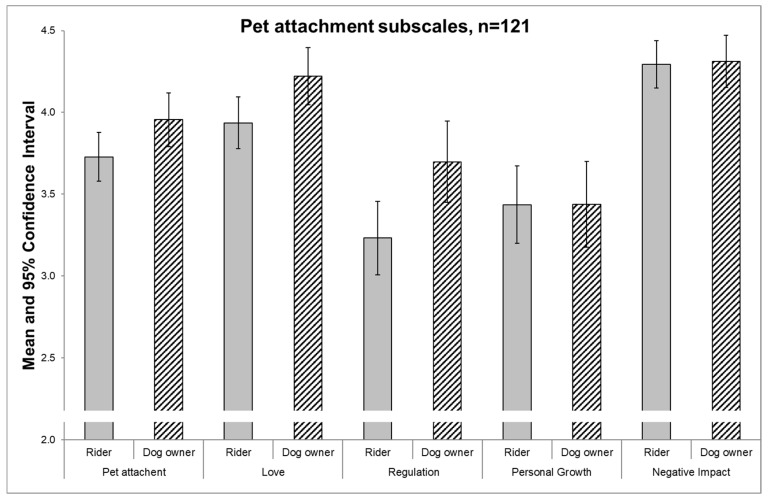
Pet attachment and subscales scores (mean and 95% confidence intervals) in riders and dog owners.

The first objective of the study was the evaluation of pet attachment within the two groups and possible gender differences. Dog owners reported no significantly higher attachment to their pets (3.91 +/− 0.613) compared to riders (3.76 +/− 0.637) (*p* = 0.053) (Figure 1). Covariates age, sex, BMI, marital status and educational status did not confound the results.

Recreational horseback riders show significantly (*p* = 0.023) lower pet attachment “love” scores (3.94 +/− 0.08 SEM) compared to dog owners (mean 4.22 +/− 0.09 SEM). “Regulation of emotions” was also higher (*p* = 0.009) in dog owners (3.69 +/− 0.13 SEM) compared to recreational horseback riders (3.23 +/− 0.11 SEM). No significant differences were found for “personal growth” (3.44 +/− 0.12 SEM) compared to dog owners (3.44 +/− 0.13 SEM) (*p* = 0.994) and “negative impact” (riders: 4.29 +/− 0.07 SEM, dog owners: 4.31 +/− 0.08 SEM) (*p* = 0.869) (Table 2).

### 3.3. Gender Differences

The evaluation of overall pet attachment shows no significant difference between female riders (mean 3.8 +/− 0.1 SEM) and male riders (mean 3.4 +/− 0.2 SEM) (*p* = 0.080). Also, female dog owners (mean 3.9 +/− 0.1 SEM) and male dog owners (mean 3.9 +/− 0.1 SEM) did not differ significantly (*p* = 0.990).

**Figure 2:**
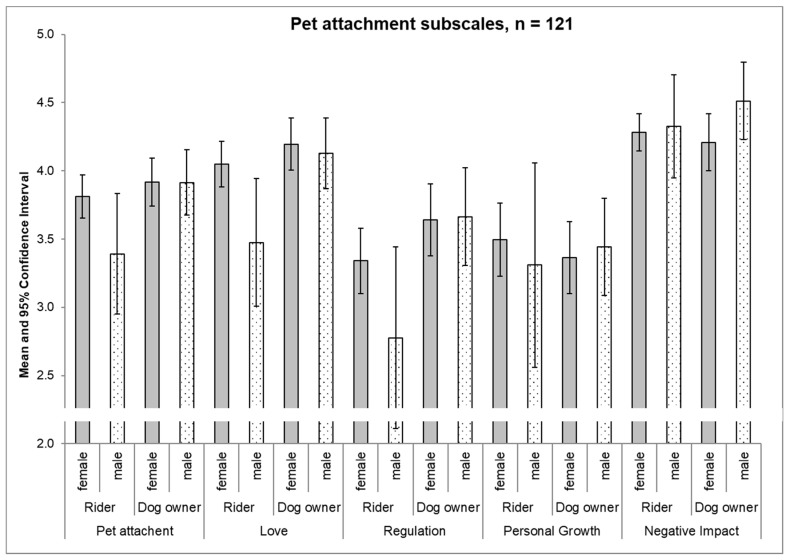
Pet attachment and subscales scores of riders and dog owners (mean and 95% confidence intervals) by sex.

However, scores for the “love” factor showed higher (*p* = 0.025) values in female riders (4.0 +/− 0.1 SEM) than in male riders (3.5 +/− 0.2 SEM), while female dog owners (4.2 +/− 0.1 SEM) did not differ significantly from male dog owners (4.1 +/− 0.1 SEM) (*p* = 0.698). All other subscales showed no statistically significant differences between males and females, neither in riders nor in dog owners (Figure 2).

### 3.4. Pet Attachment and Wellbeing

To answer the second research question, pet attachment scores (PALS-35 questionnaire) were correlated with physical, psychological and social wellbeing scores assessed for the time periods during and after the activity. In addition to these evaluations we assessed the association between pet attachment scores and overall wellbeing FAHW 12-scores. Neither in riders (*R* = 0.142, *p* = 0.251), nor in dog owners (*R* = 0.091, *p* = 0.507 such a relationship prevailed.

#### 3.4.1. Physical Wellbeing

We observed no correlation of physical wellbeing in riders and dog owners (for the assessment periods during and after the activity with their animal) and overall pet attachment (during: riders: *R* = 0.120, *p* = 0.334; dog owners: *R* = 0.162, *p* = 0.229; after: riders: *R*= −0.007, *p* = 0.955; dog owners: *R* = 0.103, *p* = 0.445). Also, no correlation of physical wellbeing with the pet “love” factor, “regulation” factor, “personal growth” and “negative impact” factors in riders and dog owners were found for the period during the activity (Table 3). Also no correlation was found between the FAHW 12-scale (rider: *R* = 0.123, *p* = 0.320; dog owner: *R* = 0.200, *p* = 0.140) and pet attachment.

#### 3.4.2. Psychological Wellbeing

Psychological wellbeing during the activity and overall pet attachment showed significant correlations in both groups (riders: *R* = 0.350, *p* = 0.004, dog owners: *R* = 0.246, *p* = 0.065). Furthermore, a correlation of psychological wellbeing and the “love” factor during the activity could be demonstrated in riders (riders *R* = 0.283, *p* = 0.020) but not in dog owners (dog owners: *R* = 0.211, *p* = 0.115). Similarly, the “regulation” factor was correlated with psychological wellbeing in riders (*R* = 0.345, *p*=.004), but not in dog owners (*R* = 0.178, *p* = 0.184). “Personal growth” shows a correlation with psychological wellbeing during the activity in riders and dog owners (riders: *R* = 0.317, *p* = 0.009; dog owners: *R* = 0.288, *p* = 0.030), the “negative impact” factor did not correlate in either group (Table 4). Pet attachment did not correlate with psychological wellbeing subscale of the FAHW 12-scale (riders: *R* = 0.207, *p* = 0.093; dog owners: *R* = 0.109, *p* = 0.426).

#### 3.4.3. Social Wellbeing

In both groups (riders: *R* = 0.413, *p* = 0.001; dog owners: *R* = 0.407, *p* = 0.002) a correlation between pet attachment and social wellbeing perceived for the time period during the activity was found. Social wellbeing was correlated with the pet attachment “love” factor (riders: *R* = 0.374, *p* = 0.002, dog owners: *R* = 0.411, *p* = 0.001), with the “regulation of emotions” factor (riders: *R* = 0.437, *p* < 0.001; dog owners: *R* = 0.344, *p* = 0.009) and the “personal growth” factor (riders: *R* = 0.282, *p* = 0.021, dog owners: *R* = 0.421, *p* = 0.001). The factors “negative impact” and social wellbeing were not correlated neither in riders nor in dog owners (Table 5). Also pet attachment did not correlate with the social wellbeing subscale of the FAHW 12-scale (riders: *R* = 0.088, *p* = 0.479; dog owners: *R* = 0.035, *p* = 0.798).

For the assessment period “after the activity” neither a correlation of physical nor of psychological and social wellbeing and pet attachment in horseback riders and dog owners was found (Table 3, Table 4 and Table 5).

### 3.5. Correlation Pet Attachment and Mood

Overall pet attachment and mood state during/after the activity with the animal were correlated in both groups (riders: *R* = 0.331, *p* = 0.006; dog owners: *R* = 450, *p* = 0.001), and also the “love” factor (riders: *R* = 387, *p* = 0.001; dog owners: *R* = 414, *p* = 0.002) was correlated with mood. No statistically significant correlation of mood and the “regulation” factor was observed in horseback riders (*R* = 208, *p* = 0.091), but in dog owners (*R* = 386, *p* = 0.004)). A correlation with the “personal growth” factor was seen in riders (*R* = 248, *p* = 0.043) and in dog owners (*R* = 422, *p* = 0.001). We found no correlation of mood with the factor “negative impact” in neither group (Table 6).

## 4. Discussion

This is the first study investigating pet attachment and self-reported wellbeing of middle and older-aged (45+) recreational horseback riders and comparing the results with those of dog owners.

We hypothesized that recreational horseback riders, regardless of gender, show comparable pet attachment scores compared to dog owners and that pet attachment of recreational horseback riders is associated with their physical, psychological and social wellbeing.

We demonstrated that dog owners reported somewhat higher attachment to pets compared to recreational horseback riders, but this difference did not reach statistical significance. The results of Smolkovic [24] showed highest attachment to dogs and also Winefield [25], Raina [10] and Mein and Grant [23] reported highest attachment to dogs in older people, but horseback riders were not investigated. In the category very high attachment to their pets, dog owners (78%) occupy the first place, followed by cat owners (64%) [23]. Pet ownership has been found to be generally related to high levels of attachment across a number of studies [11,13,20,26].

The present study shows higher attachment scores for all participants compared to the results of Cromer et al, who developed the PALS-35-questionnaire, with only “negative impact” being approximately equal to the results of this study [46]. Recreational horseback riders reported higher values for all four factors compared to dog owners in Cromer’s study. Also, the “love” factor in horseback riders showed higher scores compared to dog owners, cat owners and owners of other animals in Cromers’s study [46]. Cat ownership [46] was associated with lower “negative impact” compared to horseback riders and dog owners of the present study. Cromer and Barlow performed their study with a sample of college students with a mean age of 19 years [46]. The differences to our results could thus be due to the higher age of our study participants. Middle and older-aged people, perhaps living alone, are likely to care more for their dog or for their horse and feel responsible for them.

The detailed evaluation of pet attachment shows significantly lower “love” scores and “regulation of emotions” of recreational horseback riders compared to dog owners (Table 2). This finding could be due to the living circumstances. Companion dogs live with their owners in the house, the dogs sleep with their owners, which is something horse owners could not do. The difference in proximity to the animal may explain the difference in emotional closeness. Furthermore, dog owners may have contact to their dogs the whole day, which is not the case in riders. We found no significantly lower values for “personal growth benefits” and “negative impact” (Table 2). When living with a dog, caring for the dog [50] or illness of the dog can lead to stressful situations. Caring for a horse, feeding it or giving medications is typically done by stable workers and not by horse owners, but they also feel responsible for their horse and are concerned about the situation.

Similar to other studies [51], we found a significant correlation of self-reported mood during the activity with the animal and pet attachment, with pet love and personal growth in both groups being particularly strongly associated (Table 6). People love the activity with their pets. Therefore, positive emotions [51] and happiness [10] increase.

### 4.1. Gender Differences

Earlier studies indicate gender differences in pet attachment [13,24,25,46,52]. We observed gender differences in the “love factor”, with female riders reporting significantly higher values of attachment than men, but no significantly higher overall pet attachment of female dog owners compared to males was found (Figure 2). While emotional attachment to pets is most apparent in the “love” factor, and the differences between males and females are consistent with the literature in riders, the failure to detect gender differences in dog owners could be due to the higher age of our study groups. Female dog owners and male dog owners had higher values for “regulation of emotions” compared to riders. As mentioned earlier, dog owners live close to their dogs and benefit from their animal every day, sometimes the whole day. Most riders live in distance from their horse and do not see it daily.

### 4.2. Relation to Wellbeing

The second objective of the study was to assess whether pet attachment is associated with physical, psychological and social wellbeing.

It has been implicated previously that pet attachment is associated with physical [21,26,32] psychological [21,27,36] and social [36,37,53] health benefits. We partially support these results since we found significant correlations between pet attachment and psychological and social wellbeing.

Psychological wellbeing for the assessment period during the activity was significantly correlated with overall pet attachment in riders (Table 4), which is in agreement with results about service dog ownership [54]. Also in therapeutical settings, pets are used for emotional regulation [46]. People feel positive emotions [51], also love [27], due to the contact with animals. Riding a horse needs continuous concentration on the animal to be able to get involved with the horse and the situation. This concentration can lead to reduced psychological stress.

Also the “personal growth” factor was significantly correlated with psychological wellbeing, both in riders and dog owners, which is in agreement with previous findings [53]. Both horses and dogs are strong animals. Being able to guide them can strengthen self-confidence.

For “negative impact” no correlation with psychological wellbeing was observed. However, it is clear from other studies, that pet loss [36,55], providing for the animal [50], and illness of the animal can lead to stress, symptoms of depression [22] and other adverse experiences of their owners [56]. Older-aged people might have experienced many such events during the course of their life and have learnt to cope with it.

Social wellbeing during the activity and pet attachment “love” factor, “regulation” factor and “personal growth” correlated significantly in both groups (Table 5). Both, recreational horseback riders and dog owners, have to leave their home, they may meet friends and come in contact with different people [36,37,40] improving quality and quantity of social contacts. 

In contrast to Gadomski et al [32] and Shintani [54] we found no correlation of physical wellbeing during and after the activity with overall pet attachment. These studies were conducted in service dog owners, who experience improvements of diverse abilities due to the animal, or investigated children that have to be considered as a specific group with specific benefits from interaction with animals. 

### 4.3. Limitations of the Study

The cross-sectional design of the study prohibits making causal inferences. Although the applied questionnaire is a tested approach for people in the chosen age range, it is possible that a minority of older-aged participants would need support in answering the questions. We did not measure biomarkers, such as serum cortisol, blood pressure or heart rate variability for the evaluation of stress. We used the self-reported questionnaire also for assessing the influence on subjective wellbeing. Therefore, no objective measurements were available for physical health. Future research could benefit from using a combination of self-reporting and observational methods to reduce the possibility of bias.

Nevertheless, the fact that in elderly people achievement of social interaction is difficult but can obviously be reached through the additional activity with animals should be considered as a means to improve social contacts in older people. It seems that recreational horseback riding provides similar benefits as is already known for dog owners.

## 5. Conclusions

Middle and older-aged recreational horseback riders and dog owners profit especially concerning psychological and social wellbeing due to their pet attachment. Consistent with our hypothesis that emotional relationship to an animal improves wellbeing, we found a significant correlation of mood during/after the activity with overall pet attachment and its sub-scales. This could be due to positive emotions induced by the activities with the pets. Similarly, psychological wellbeing during the activity was significantly correlated with overall pet attachment in riders, and a significant correlation of pet attachment and social wellbeing during the activity in both, riders and dog owners, was observed.

This study adds additional evidence and extends existing knowledge about pet attachment. Regardless of whether dogs or horses are involved, pet ownership strongly supports the assumption that activity with and closeness to these animals promotes subjective wellbeing.

## Figures and Tables

**Table 1:** Demographic characteristics of all participants and according to study groups.

Demographic Characteristics	All Participants	Riders	Dog Owners	*p*-Value
Participants, *n*	124	67	57	
Males	29 (23.4%)	8 (11.9%)	21 (36.8%)	0.001 x^2^
Age mean +/− SD in years	56.94+/− 9.3	55.5 +/− 8.4	58.7 +/− 10.1	0.090 MW
Age Range	45–82	45–82	45–80	
Fixed partnership in %	78.20%	76.10%	80.70%	0.538 x^2^
Educational status (≥12 years)	92.70%	92.50%	93.00%	0.868 x^2^
Academic degree	30.60%	37.30%	22.80%	0.010 x^2^

*n*, number; SD, standard deviation; %, percent, x^2^, chi square-test; MW, Mann-Whitney U-test.

**Table 2:** Results of general linear model for pet attachment subscales. Regression coefficients representing the adjusted mean difference to dog owners and their 95% confidence intervals and associated *p*-values are shown.

Pet Attachment Subscale	Riders Compared to Dog Owners			
	Adjusted Mean Difference	95% CI		*p*-Value
Love	−0.285	−0.529	−0.040	0.023
Regulation	−0.466	−0.815	−0.117	0.009
Personal Growth	−0.001	−0.369	0.366	0.994
Negative Impact	−0.019	−0.241	0.204	0.869

CI: 95%Confidence interval. *p*: *p*-value for test for zero difference.

**Table 3:** Spearman correlation of physical wellbeing and pet attachment scores.

Physical Wellbeing	Pet Attachment Scores	Riders		Dog Owners	
		*Correlation Coefficient*	*p*-Value	*Correlation Coefficient*	*p*-Value
Wellbeing during	Pet attachment	0.120	0.334	0.162	0.229
	Pet attachment love	0.092	0.459	0.047	0.730
	Pet attachment regulation	0.105	0.400	0.149	0.269
	Pet attachment personal growth	0.123	0.322	0.153	0.255
	Pet attachment negative Impact	0.082	0.509	0.085	0.527
Wellbeing after	Pet attachment	0.007	0.955	0.103	0.445
	Pet attachment love	0.008	0.947	0.048	0.721
	Pet attachment regulation	0.047	0.707	0.098	0.468
	Pet attachment personal growth	−0.035	0.778	0.186	0.165
	Pet attachment negative impact	0.057	0.647	−0.133	0.323

**Table 4:** Spearman correlation of psychological wellbeing and pet attachment scores.

Psychological Wellbeing	Pet Attachment Scores	Riders		Dog Owners	
		*Correlation Coefficient*	*p*-Value	*Correlation Coefficient*	*p*-Value
Wellbeing during	Pet attachment	0.350	0.004	0.246	0.065
	Pet attachment love	0.283	0.020	0.211	0.115
	Pet attachment regulation	0.345	0.004	0.178	0.184
	Pet attachment personal growth	0.317	0.009	0.288	0.030
	Pet attachment negative Impact	−0.021	0.864	0.027	0.844
Wellbeing after	Pet attachment	0.171	0.166	0.173	0.198
	Pet attachment love	0.156	0.206	0.190	0.158
	Pet attachment regulation	0.176	0.154	0.146	0.279
	Pet attachment personal growth	0.052	0.679	0.215	0.108
	Pet attachment negative impact	0.110	0.375	−0.200	0.136

**Table 5:** Spearman correlation of social wellbeing and pet attachment scores.

Social Wellbeing	Pet Attachment Scores	Riders		Dog Owners	
		*Correlation Coefficient*	*p*-Value	*Correlation Coefficient*	*p*-Value
Wellbeing during	Pet attachment	0.413	0.001	0.407	0.002
	Pet attachment love	0.374	0.002	0.411	0.001
	Pet attachment regulation	0.437	0.000	0.344	0.009
	Pet attachment personal growth	0.282	0.021	0.421	0.001
	Pet attachment negative Impact	−0.115	0.352	−0.068	0.614
Wellbeing after	Pet attachment	0.210	0.089	0.205	0.125
	Pet attachment love	0.216	0.079	0.247	0.064
	Pet attachment regulation	0.167	0.177	0.163	0.226
	Pet attachment personal growth	0.137	0.268	0.243	0.068
	Pet attachment negative impact	−0.063	0.611	−0.213	0.111

**Table 6:** Spearman correlation of mood estimation and pet attachment scores.

Pet Attachment Scale	All Participants		Riders		Dog Owners	
	*Correlation Coefficient*	*p*-Value	*Correlation Coefficient*	*p*-Value	*Correlation Coefficient*	*p*-Value
Overall pet attachment	0.366	≤0.001	0.331	0.006	0.450	0.001
Love	0.383	≤0.001	0.387	0.001	0.414	0.002
Regulation	0.262	0.004	0.208	0.091	0.386	0.004
Personal growth	0.332	≤0.001	0.248	0.043	0.422	0.001
Negative impact	0.063	0.496	0.078	0.530	0.084	0.545

## Supplementary Materials

The following are available online at https://www.mdpi.com/1660-4601/17/6/1865/s1, S1: Questionnaire.doc.
