# Supplementary material for: Pet Attachment and Wellbeing of Older-Aged Recreational Horseback Riders
Source: Int J Environ Res Public Health. 2020 Mar 13;17(6):1865. doi: 10.3390/ijerph17061865 (PMC7143422; doi:10.3390/ijerph17061865)

How do you usually feel during/after riding?

Please mark with a cross where applicable and state why you agree.

	**During riding / dog playing or walking**	What do you think are the reasons? *Enter manually*	**After riding / dog playing or walking**	What do you think are the reasons? *Enter manually*
1. Overall relaxed	O		O	
2. Physical fit	O		O	
3. I forget my pain	O		O	
4. Entirely well	O		O	
5. In harmony with the horse/dog	O		O	
6. Physically tired	O		O	
7. I forget my worries	O		O	
8. Stressed	O		O	
9. Mentally fit	O		O	
10. Have high pulse rate	O		O	
11. Mental tired	O		O	
12. Mental relaxed	O		O	
13. Assertive	O		O	
14. I'm full of energy	O		O	
15. Understood	O		O	
16. I feel pain	O		O	
17. Tensed	O		O	
18. I feel energy	O		O	
19. I only think about riding/playing with the dog	O		O	
20. I feel inferior	O		O	
21. Strong	O		O	
22. Motivated to achieve more	O		O	
23. Exhausted	O		O	
24. Safe	O		O	
25. Breathless	O		O	
26. Accepted by the others	O		O	
27. Relieved because I managed the challenge	O		O	
28. Sad	O		O	

Which face illustrates best how you felt during and feel now after riding/dog playing or dog walking?

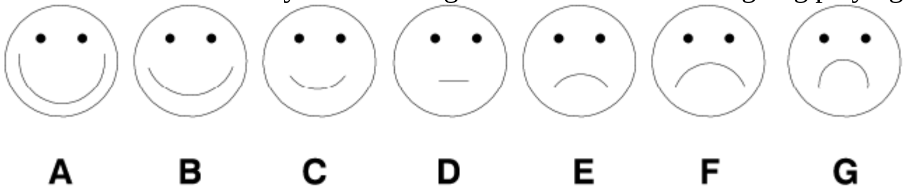

Supplement: Supplementary file 1 [file ijerph-17-01865-s001.pdf]
